# Adaptive radiotherapy in locally advanced head and neck cancer: The importance of reduced margins

**DOI:** 10.1016/j.phro.2025.100696

**Published:** 2025-01-11

**Authors:** Hedda Enocson, André Haraldsson, Per Engström, Sofie Ceberg, Maria Gebre-Medhin, Gabriel Adrian, Per Munck af Rosenschöld

**Affiliations:** a Medical Radiation Physics, Department of Clinical Sciences Lund, Lund University, Lund, Sweden; b Radiation Physics, Department of Hematology, Oncology, and Radiation Physics, Skåne University Hospital, Sweden; c Oncology, Department of Clinical Sciences Lund, Lund University, Lund, Sweden; d Oncology, Department of Hematology, Oncology, and Radiation Physics, Skåne University Hospital, Sweden

**Keywords:** Adaptive radiotherapy, Head-and-neck cancer, Re-planning, Radiotherapy, Tomotherapy, VMAT, Deformable registration, IGRT

## Abstract

**Background and Purpose:**

Adaptive radiotherapy (ART) involves treatment re-planning based on anatomical changes, which may improve target coverage and sparing of organs-at-risk (OARs). This study retrospectively assessed the technical feasibility and potential benefits of daily ART in combination with reduced planning target volume (PTV) margins for head and neck squamous cell carcinoma (HNSCC).

**Materials and Methods:**

Thirty-one patients, encompassing 902 treatment fractions, treated with radiotherapy to 60.0–68.0 Gy in 2 Gy/fraction were studied. Synthetic CTs (sCT) from daily kVCT images were created and contours propagated using deformable image registration (DIR). Target contours were reviewed and corrected. On the sCT, non-adapted delivered doses and ART-plans with 5 mm (clinical standard) and 2 mm PTV-margin were evaluated. All daily dose distributions were then accumulated.

**Results:**

Target contours required correction in 48 % of the fractions. Daily non-adapted D_98%,CTV_ was > 95 % in 890 (5 mm) and 825 (2 mm) out of 902 fractions. All adapted plans achieved D_98%,CTV_ > 95 %. Significant reductions in mean doses to OARs were observed for PTV = 2 mm ART-plans: 4.1 Gy for parotid, 2.6 Gy for submandibular, 3.3 Gy for oral cavity, 4.0 Gy for esophagus, and 3.8 Gy for larynx.

**Conclusion:**

ART-planning on sCT and DIR propagated contours was feasible and promising for further clinical testing. To obtain a potential clinical benefit of ART, a synchronous reduction of the PTV-margin was warranted. Daily ART can be used to maintain adequate target dosimetry for every fraction, though for the accumulated treatment, insufficient target coverage without ART is unlikely to occur.

## Highlights

• Daily imaging was used to assess adaptive radiotherapy for head and neck cancer.
• Plan adaptation on synthetic CT and deformed contours was clinically feasible.
• With 2 mm margin, 16/31 of patients and 77/902 fractions needed plan adaptation.
• Adaptation with smaller margins was required to reduced doses to organs at risk.

## 1 Introduction

Adaptive radiotherapy (ART) is a method for continuous adjustment of treatment based on anatomical and/or biological changes. With ART, the treatment plan is geometrically changed due to anatomical variations such as weight loss, tumor response, bladder and bowel filling [1]. Biological ART refers to changing the treatment based on bio- markers such as PSA changes, tumor response etc. [2], [3]. This study focused on using ART for anatomical inter-fractional changes. ART can be performed using different strategies: Scheduled or ad-hoc offline ART between fractions, or online ART with the patient in-room [4]. Offline ART can be based on new simulation CTs or verification images acquired during treatment. Online techniques involve fast re-planning on the daily acquired verification image, typically a deformed synthetic CBCT [5] or an MR image from an MR-linac [4], with the patient on the couch before treatment delivery. Studies have reported shrinkage of the primary clinical target volume (CTV-T) ranging from 6-69 % by the end of fractionated radiotherapy (RT) for patients with head and neck squamous cell carcinoma (HNSCC) [6], [7], [8], [9], [10], [11], [12]. The involved lymph nodes (CTV-N) have been shown to shrink to a similar degree [7], [11].

Setup errors correlate with changes in body weight for HNSCC patients undergoing RT [13]. Anatomical changes during RT can cause hot and/or cold spots in the target volume [8], [9], [14], [15] while other authors have observed no changes in target coverage despite anatomical changes [7]. The parotid glands have been reported not only to shrink throughout treatment but also migrate closer to the high dose region [8], [9], [12], [15] resulting in elevated dose and consequently an increased risk of xerostomia [12], [14], [16]. Differences in mean parotid dose between planning CT and repeated CT scans, have been reported up to 10 Gy, while other studies have observed minor dosimetric effects (<1 Gy) despite significant anatomical changes [16].

A recent daily ART simulation study of 20 patients showed significantly improved planning target volume (PTV) coverage and lowered spinal cord doses compared with no-ART [17]. One to three re-plans through the treatment course have been shown to reduce the mean parotid dose by 0–10 Gy [7], [12], [18], [19], [20], [21] and the maximum spinal cord dose by 0.5–6.7 Gy [19], [20]. The variability in these dose reductions may relate to the variability in prescribed doses, which ranged from 60 to 76 Gy. Reducing PTV-margins spare mean doses to organs at risk (OAR) by approximately 1 Gy/mm but at the compromise of target coverage, yet one or two re-plans have been shown to improve target coverage, even in plans with 0 mm margin [7], [22].

A recent systematic review of 15 prospective trials on ART for HNSCC showed reduced doses to the parotid glands and spinal cord, with one to two re-plans per patient and PTV-margins from 0 to 5 mm (median 20 patients per study, range 4–86) [20]. Only two studies reported on clinical outcome and reported improvements on toxicity were none or modest. In the ARTIX trial, a phase 3 randomized trial of 132 oropharyngeal cancer patients, ART did not significantly improve patient-reported outcomes, or rates of toxic effects compared with standard radiotherapy [23]. Existing literature presents a wide range of findings regarding the efficacy of ART in HNSCC, with variable number of adaptations, PTV-margins, and ART strategies. The conclusions on benefits with ART for HNSCC are inconsistent, likely due to small study cohorts and the variability in target configuration [6]. Simulated workflow times for ART treatments using a simultaneous integrated boost (SIB) technique have been reported to be 30 min on average, excluding image acquisition and treatment delivery [17]. As re-planning is a labor-intensive and time-consuming process, identifying the most beneficial strategy with the least required re-plans is of interest.

Our hypothesis is that (1) daily ART is probably not warranted to achieve good dosimetry for all patients and risk becoming too labor-intensive, and (2) without PTV-margin reduction, the benefits of ART for HNSCC patients may not justify the resources needed. Therefore, we conducted a retrospective study with the aim to evaluate the technical feasibility and dosimetric benefits of daily ART in HNSCC. Specifically, we investigated if reducing the PTV-margins to 2 mm could enhance OAR sparing while maintaining adequate target coverage. Both non-adaptive and adaptive radiotherapy dosimetry was simulated on daily images and was accumulated for the entire treatment. To our knowledge, this study is the first to assess the impact of PTV-margins on daily and accumulated ART distributions calculated from non-deformed verification fan-beam CT images.

## 2 Materials and methods

### 2.1 Patients

A consecutive cohort of 31 HNSCC patients (902 treatment fractions) were retrospectively studied (Table 1), all previously treated at Skåne University Hospital (Lund, Sweden) with helical tomotherapy (HT) on a CT-linac (Radixact®, Accuray, Madison, WI) between August 2022 and April 2023. All patients were treated with curative intent, without distant metastases, and none were re-planned during treatment. Patients were prescribed 60.0–68.0 Gy, in 2.0 Gy/fraction to the primary target volume (PTV-T) and positive nodal volumes (PTV-N) and 50.0–54.4 Gy to elective volumes using SIB technique. The treatment was performed with a 5 mm CTV-PTV-margin and daily fan beam kVCT (ClearRT™, Accuray, Madison, WI) imaging. Patient data collection and analysis was approved by the ethics committee (Dnr 2020–04164, Stockholm, Sweden).

**Table 1:** Study cohort and treatment characteristics.

**Patient Characteristics**	N	%
**Total**	31	100
Age (years)		
Median (range)	65 (41–78)	
>65	14	45
<=65	17	55
Sex		
Female	9	29
Male	22	71
BMI		
Median (range)	28.01 (20.16–31.94)	
Primary tumor site		
Oral cancer	7	23
Oropharynx	15	48
Salivary glands	4	13
Larynx	3	10
Hypopharynx	1	3
CUP	1	3
T stage		
T0	2	6
T1	1	3
T2	14	45
T3	6	19
T4	8	26
Nodal Status		
N0	5	16
N1	14	45
N2	5	16
N2b	2	6
N2c	4	13
N3b	1	3
Recurrence	1	3
p16*		
Positive	15	48
Negative	1	3
Surgery (Reconstructive)**	13 (5)	42 (16)
Concomitant chemotherapy	8	26
Prescribed dose (Gy)		
68.0	20	65
60.0–66.0	11	35
Reirradiation	0	0
Weight change during RT (kg)		
Median (range)	−2.4 (−8.4–2.1)	
Primary GTV volume (cm^3^)		
Median (range)	5.55 (0–61.06)	
CTV volume (cm^3^)		
Median (range)	284.8 (40.3–666.5)	

* p16 only relevant for oropharyngeal cancer and CUP. **Reconstructive surgery include muscle and/or skin transfer procedures. BMI; Body mass index. CUP; Cancer of unknown primary. CTV; Clinical target volume.

### 2.2 Synthetic CT generation and contour propagation

A synthetic CT (sCT) was generated from the daily kVCT images with deformable image registration (DIR) (RayStation 2024A DTK, RaySearch Laboratories, Stockholm) between the planning CT (pCT) and the kVCT. Dose calculation on kVCT images has previously shown to be feasible [24]. Here, the sCT was primarily generated to rectify missing field of view (FoV) by filling in the omitted volumes around the target, which was always within the FoV, and to correct the HU-values to match the CT calibration curve used for the pCT.

**Fig. 1:**
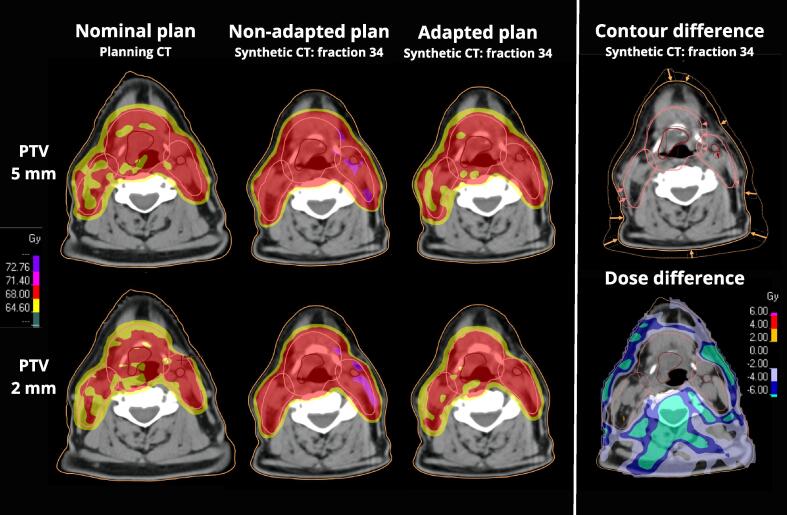
Dose distributions of nominal plan on planning CT, non-adapted and adapted plan calculated on daily synthetic CT at last treatment fraction, for 5 mm and 2 mm PTV-CTV margin respectively. The contour difference illustrates the anatomical changes in GTV (red), CTV (pink) and BODY (orange) contours where the dashed lines are the planning contours on the planning CT and the solid lines are the deformed contours on the at the last treatment’s synthetic CT. Arrows highlight the difference between contours. Dose difference visualize the difference in dose distribution between 2 mm and 5 mm plan. Patient with the highest reported weight loss chosen as an example. (For interpretation of the references to colour in this figure legend, the reader is referred to the web version of this article.)

A DIR method in a novel research version in RayStation was used to propagate the GTV/CTV and OAR contours onto the daily sCT. The OARs were propagated using DIR over the whole image. The GTV-N and CTV volumes were propagated using DIR focused on a region expanded 1 cm around the respective structure. Since correct delineation of the primary tumor volume could not be ensured on the sCT images, the GTV-T was propagated using a rigid image registration in a region expanded 2 cm around the structure (Fig. 1). Artifacts were handled according to clinic standards, contoured on the pCT, and deformed to the sCT with a density set to water. The DIR algorithm has previously been described and validated for HNSCC patients [25], [26], [27], [28], [29], [30]. The propagated structures were controlled and graded by radiation oncologist as; 1. Clinically Acceptable (No Modification Needed), 2. Acceptable with Minor Modification (Less than 2 mm), 3. Unacceptable with Major Modification (Greater than 2 mm). The structures were manually adjusted if the changes were considered grade 3. The DIR propagation was also validated according to the AAPM report “Use of image registration and fusion algorithms and techniques in radiotherapy” [31] using virtual phantoms generated in ImSimQA™ software (Oncology System Limited, UK). The propagated structures were evaluated against the virtual phantoms' ground truth using Dice similarity coefficient (DSC), mean and max distance to agreement (DTA).

### 2.3 Treatment simulation and dosimetric evaluation

Additional plans with 2 mm CTV-PTV-margin were created, resulting in two nominal plans for each pCT. The nominal plans, with 5 and 2 mm CTV-PTV-margin, were re-calculated onto the daily sCT images to obtain the estimated non-adapted delivered dose. Adaptive plans were generated on the daily sCT images using the same optimization conditions as the nominal plans (Fig. 1). The daily dose distributions were deformed to the pCT, and the total no-ART and ART doses were accumulated for the treatment plans using 5 and 2 mm PTV-margin, respectively.

Normal Tissue Complication Probability (NTCP) was calculated for OARs and endpoints; parotid: xerostomia, spinal cord: necrosis, larynx: cartilage necrosis, esophagus: acute esophagitis [32].

To evaluate the necessity of adaptation in ensuring CTV coverage, the accumulated and fraction doses using both the 5 and 2 mm PTV-margin, were compared. Adaption criteria was defined as: intervention required when the daily dose to 98 % of the CTV (D_98%,CTV_) < 95 %, which was consistent with planning criterion for PTV at our department.

### 2.4 Statistics

Wilcoxon Signed Rank Test assessed the difference in dose between non-adapted 5 mm plans and adapted 5 mm plans, non-adapted 2 mm plans and adapted 2 mm plans for target and OAR volumes. Chi-squared test was used to evaluate the relationship between the number of fractions where D_98%,CTV_ < 95 % and categorical patient characteristics, assuming that observations were independent. The Fisher test was utilized to determine the relationship between number of fractions where D_98%,CTV_ < 95 % and primary tumor site. Spearman Correlation assessed any correlation between CTV-T grading and the continuous patient characteristics. The corresponding functions wilcoxon, chi2_contingency, spearmanr, and fisher_exact from the scipy.stats module in Python 3.11.9 were employed to conduct these statistical tests. In this exploratory analysis, we consider p-values of less than 0.05 to be statistically significant.

## 3 Results

Generating synthetic CTs was feasible for all patients, with satisfactory soft-tissue image quality for contouring in all but one case. Apart from this case, contour propagation was feasible for all patients. In 48.4 % of the cases, patients required manual corrections of the CTV contour, particularly those with dental artifacts and altered air cavities for tumors abutting the oral cavity and trachea. Specifically, the CTV-T, CTV-N and elective CTV required recontouring in 46.7 %, 6.2 % and 13.6 % of the fractions. The quality of propagated contours was graded; see Supplementary Figs. 5 and 6. The deformation verification with virtual phantom showed a mean DSC higher than 0.95 for CTV contours; see Supplementary Table 4.

**Fig. 2:**
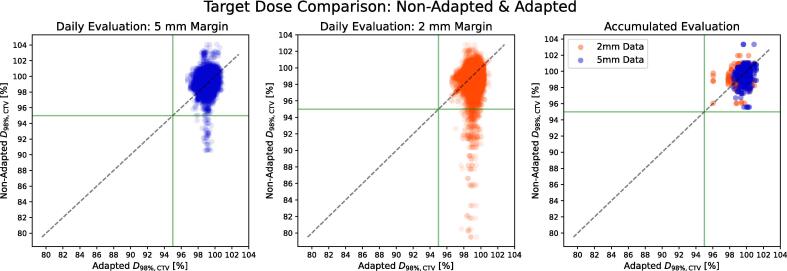
Fractional and accumulated evaluation of D_98%,CTV_ for non-adapted and adapted doses for 5 mm (blue) and 2 mm (orange) PTV margins respectively. The green line marks the intervention threshold of 95 % of the prescribed dose and the black dashed line is the identity (X = Y). (For interpretation of the references to colour in this figure legend, the reader is referred to the web version of this article.)

Daily non-adapted plans had D_98%,CTV_ > 95 % in 890 and 825 out of the total 902 fractions for the 5 and 2 mm PTV-margin plans, respectively (Fig. 2). For the combined CTV, we found that 1.3 % (12 fractions) and 7.8 % (77 fractions) of the non-adapted fractions had D_98%,CTV_ < 95 %, and for 4 and 16 out of the 31 patients had one or more fractions had D_98%,CTV_ < 95 %, for the 5 and 2 mm PTV-margin plans respectively. Looking specifically at CTV-T, 0.8 % (7 fractions) and 5.0 % (45 fractions) had D_98%,CTV-T_ < 95 %, for CTV-N, 0.7 % (5 fractions) and 1.2 % (8 fractions) had D_98%,CTV-N_ < 95 % and for the elective CTV 0.3 % (2 fractions) and 5.2 % (35 fractions) had D_98%,CTV, elective_ < 95 %.

**Fig. 3:**
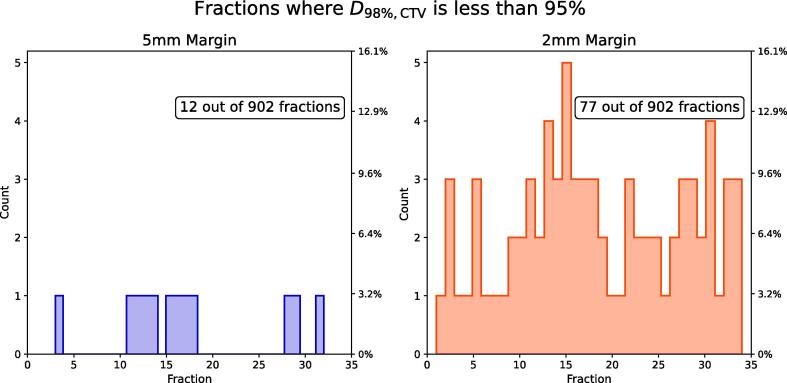
Distribution of the 12 and 77 events where D_98%,CTV_  < 95 % over the treatment course for 5 mm (blue) and 2 mm (orange) plans respectively. (For interpretation of the references to colour in this figure legend, the reader is referred to the web version of this article.)

The distribution of events, where one or more CTVs per patients had D_98%,CTV_ < 95 % over the treatment course, showed no obvious trends (Fig. 3). In adapted plans, all fractions had D_98%,CTV_ > 95 %, both regarding the 5 and 2 mm PTV-margin plans.

The accumulated D_98%,CTV_ was greater than 95 % in all cases, both for 5 and 2 mm PTV-margin and with/without adaptation. No significant differences in D_98%,CTV_ were observed between the accumulated non-adapted 5 mm, non-adapted 2 mm and adapted 2 mm PTV-margin plans (Table 3 Supplementary). Significant differences were found between the accumulated non-adapted and adapted, 5 mm margin plans (p < 0.001) for D_98%,CTV-T_ and D_98%,CTV,elective_ with median (range) difference of −0.6(−2.6 to + 1.3)% and −0.4(−1.7–2.4)% of prescribed dose, respectively. Significant difference in D_2%,CTV_ was also found for the accumulated non-adapted and adapted 5 mm margin plans (p < 0.001) with median (range) difference 0.7(−0.7–2.1)% of prescribed dose.

**Fig. 4:**
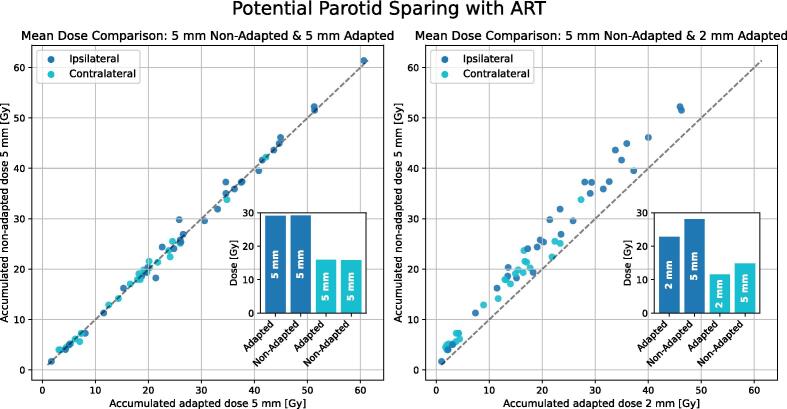
Mean dose to parotid, accumulated for 5 mm non-adapted and adapted dose distributions (left) and 2 mm adapted dose distributions (right) for the ipsi- and contra- lateral parotid. The bar plot shows the mean of each distribution.

For OARs, only insignificant dose-differences were found between non-adapted and adapted accumulated 5 mm margin plans (Fig. 4). Significant differences in the average dose to OAR were however observed between accumulated non-adapted 5 mm plans and adaptive 2 mm PTV-margin plans (Table 2). NTCP calculations showed significant difference between non-adapted 5 mm and adaptive 2 mm margin plans for parotid with endpoint xerostomia. Non-adapted 5 mm margin plans exhibited NTCP median (range) values of 24.6(0.0, 82.0)% while adaptive 2 mm margin plans offered 15.0(0.0, 73.7)%, with median (range) reductions of 9.6(0–14.8)% (p < 0.001). For the other endpoints, the NTCP calculations did not show significant differences, with the following median (range) values: for spinal cord necrosis, 0.0(0.0, 0.0)% and 0.0(0.0, 0.0)%; for larynx cartilage necrosis, 0.0(0.0, 2.1)% and 0.0(0.0, 1.8)%; and for acute esophagitis, 1.25(0.0, 52)% and 0.95(0.0, 48.3)%, for non-adapted 5 mm margin plans and adaptive 2 mm margin plans, respectively.

**Table 2:** OAR DVH values for accumulated, delivered 5 mm and adapted 2 mm plans. Values for the parotid and submandibular glands were derived from the union of the left and right volumes.

Organ at risk Median (range)	Accumulated non-adapted dose 5 mm	Accumulated adapted dose 2 mm	Difference	p-value
Mean dose [Gy]				
Parotid	22.05 (1.69–37.70)	17.89 (1.01–31.85)	4.24 (0.68–6.96)	0<.001
Submandibular	53.74 (1.87–68.64)	50.90 (1.1–67.28)	2.86 (−0.1–6.53)	0<.001
Oral cavity	40.97 (8.02–61.45)	37.13 (3.89–61.34)	3.58 (0.34–6.95)	0<.001
Esophagus	16.94 (0.32–37.61)	11.13 (0.22–33.64)	3.63 (−0.22–11.11)	0<.001
Larynx	31.88 (0.53–68.43)	27.36 (0.42–68.30)	3.45 (−2.39–10.03)	0<.001
D_2%_ [Gy]				
Spinal cord	35.14 (8.54–44.82)	27.83 (7.70–37.73)	5.33 (−1.11–13.22)	0<.001
Mandible	66.68 (25.38–69.12)	62.86 (25.21–68.74)	1.00 (−0.52–8.51)	0<.001
V_50Gy_ [cm^3^]				
Body	795.9 (179.5–1798.5)	623.8 (114.5–1541.1)	136.4 (30.5–257.4)	0<.001
V_20Gy_ [cm^3^]				
Skin	229.6 (49.5–429.4)	187.0 (26.4–381.1)	36.8 (4.0–88.6)	0<.001

The chi-square test showed a significant relationship between the number of non-adapted fractions with D_98%,CTV-T_ < 95 % and primary tumor site, with higher expected frequencies for cancer of unknown primary (CUP) and larynx (p < 0.001 and p = 0.03, for 5 and 2 mm PTV-margins, respectively). The Fisher's exact test revealed no significant difference between each primary tumor site and the remaining study cohort due to insufficient data. The Spearman test indicated a significant inverse correlation between oncologist CTV grading and the volume of the contoured image artifact in the pCT (p = 0.011), i.e. for smaller image artifact volumes a larger adjustment in target volume contours were observed.

## 4 Discussion

For the first time, a combination of fan-beam kVCT and a novel clinical DIR method shows promise for ART in HNSCC and warrant further clinical investigations. The high-quality, “CT-like”, fan-beam kVCT proved to be well suited for generation of sCTs. Our retrospective study investigated the efficacy of daily ART compared with conventional protocols using two different PTV-margins. Contrary to previous reports, where significant reductions in mean OAR doses with ART compared to no-ART protocols was found [7], [12], [18], [19], [20], our findings did not demonstrate such benefits for maintained PTV-margins. This discrepancy may help explain why the ARTIX trial [23] failed to identify any toxicity benefits associated with ART. Curiously, improvements in 2-year locoregional control and 6-month complete response with one or two re-plans versus no re-planning were reported in a non-randomized trial and a small randomized trial of 60 patients [33], [34]. Our analysis was limited to a dosimetric evaluation. However, given the small dosimetric changes observed in target dose coverage, these findings are unlikely to be supported by our cohort. Only adaptive plans with reduced margins showcased clinically relevant reductions in mean OAR doses, as seen in Table 2 for the 3 mm margin reduction, slightly surpassing previously recorded benchmarks of 1 Gy/mm [22]. When translating these dose reductions to NTCP, a significant reduction was observed only for xerostomia. Our model was based on average dose to the parotids; the risk estimate of xerostomia may be benefitted by a more sophisticated model [35].

Both plans with 5 and 2 mm PTV-margins presented occasional challenges in daily CTV dose coverage, with more frequent dose-coverage issues observed for 2 mm margin. Surprisingly, only approximately half of the patients with 2 mm PTV-margin required ART to achieve satisfactory daily CTV coverage (D_98%,CTV_ > 95 %). We were unable to find a clear prognostic marker in the current cohort that could indicate the patients most likely to benefit from ART. Though additional studies could be helpful to this end, in clinical settings, we might need to monitor and review morphological changes for all patients and intervene when required. Intervention in all fractions for all patients may be labor-intensive and with limited gains.

The frequency distribution of inferior target dose coverage over the treatment course shown in Fig. 3 suggests that ART may not be needed for each fraction using current PTV-margins. Notably, the distribution-histogram did not appear cumulative, implying that geometrical changes throughout treatment do not always persist or progress. If ART were to be used sparingly at one or two fixed instances, as many studies advocate for [18], [19], [20], [22], [36], this data suggests no obvious optimal timepoint. However, the optimal timing for re-planning has been shown to vary by OAR, with the 3rd week suggested as ideal for multiple structures [36]. A first clinical experience study on three palliative H&N patients with online ART, reported that the daily adapted plan was on average chosen in 6/13 fractions, primarily based on improved target coverage [37]. In assessing daily dose distribution, the ART plan is likely to be selected more frequently, given the magnified benefits observed in the daily distribution, which are compensated for in the dose accumulation.

Accumulating dose without ART, revealed robust CTV coverage against anatomical changes for both 5 and 2 mm plans, suggesting that ART may not be necessary to maintain accumulated dose target coverage. There were some uncertainties related to dose accumulation that may warrant further investigation, especially considering dose deformation. For dose accumulation, accurate alignment of each voxel receiving significant dose is required, whereas for contour propagation, accuracy is essential primarily at the organ boundary [31]. Uncertainties related to intra-fraction motion during the adaptation process and treatment delivery were outside the scope of this study and may need to be considered in further studies. Another consideration is that D_2%,CTV_ was significantly lower with ART while D_98%,CTV_ was higher, and from a clinical perspective whether this advantageous or not is presently unclear. Therefore, radiobiological modeling and clinical trials are required to ensure that the toxicity benefits of ART do not come at the expense of tumor control. The contour propagation process was feasible but required manual adjustments for about 50 % of the cases, underscoring the uncertainty inherent to the deformation field, with CTV-T being the most challenging. Curiously, we found an inverse correlation between image artifact volume on the planning CT and contour grading, despite artifacts impacting the contours on the synthetic CT (Fig. 6 in Supplementary). It was evident that image artifacts impact the contours, and an image artifact-reducing reconstruction could potentially improve the integrity of the deformation field. Further, a self-learning element of the contour propagation process could be beneficial, to reduce similar manual corrections on multiple treatment fractions.

Despite the ongoing search for the ideal ART strategy for HNSCC, recent surveys have found that approximately 10 % of head and neck cancer patients require replanning during treatment [38], and that 55 % of RT clinics already practice ART for HNSCC, mainly through scheduled or ad-hoc re-plans [4]. With further development of reliable and automated workflows, online ART could become a less resource-demanding re-planning alternative.

In this study we found that, while the feasibility of ART in a time- and resource-constrained clinical workflow remains unclear, the technical aspect of CT-to-kVCT contour-propagation ART was promising and warrants further clinical testing. We found that ART could benefit daily target dose coverage for HNSCC; however, the majority of treatment fractions did not require ART, even with reduced PTV margins. To achieve significant reductions in doses to OAR, it was necessary to reduce PTV margins in conjunction with ART. To maximize the clinical gain of ART for HNCC, a synchronous PTV margin reduction should be performed.

## CRediT authorship contribution statement

**Hedda Enocson:** Writing – original draft, Data curation, Formal analysis, Investigation, Methodology, Visualization. **André Haraldsson:** Writing – review & editing, Supervision, Conceptualization, Methodology. **Per Engström:** Writing – review & editing, Supervision, Methodology. **Sofie Ceberg:** Writing – review & editing, Supervision, Methodology. **Maria Gebre-Medhin:** Writing – review & editing, Supervision, Methodology, Validation. **Gabriel Adrian:** Writing – review & editing, Methodology, Validation, Validation. **Per Munck af Rosenschöld:** Writing – review & editing, Funding acquisition, Supervision, Conceptualization, Project administration, Resources.

## Declaration of competing interest

The authors declare the following financial interests/personal relationships which may be considered as potential competing interests: The authors declare that they have research agreement with Accuray Inc., Madison, WI, US and BrainLab AG, Munich, Germany.
