# Supplementary material for: Adaptive radiotherapy in locally advanced head and neck cancer: The importance of reduced margins
Source: Phys Imaging Radiat Oncol. 2025 Jan 11;33:100696. doi: 10.1016/j.phro.2025.100696 (PMC11787698; doi:10.1016/j.phro.2025.100696)
Supplement: Supplementary Data 1 [file mmc1.pdf]

Table 3. Delivered dose to CTV for 5 mm PTV margin compared with adapted 5 mm, delivered 2 mm and adapted 2 mm.

Dose-to-volume Median (range)	**Non-adapted dose 5 mm [%]**	Adapted dose 5 mm [%]	Difference [%]	Non-adapted dose 2 mm [%]	Difference [%]	Adapted dose 2 mm [%]	Difference [%]
$D_{98\%, CTV-T}$	99.08 (96.71–100.93)	99.58 (98.40–100.26)	-0.58 (-2.57–1.33) p<.001	98.77 (97.87–100.42)	0.27 (-1.60–2.22) n.s.	99.12 (97.80–99.76)	-0.03 (-2.03–1.98) n.s.
$D_{98\%, CTV-N}$	99.39 (95.58–101.05)	99.58 (98.63–100.89)	-0.14 (-4.52–1.36) n.s.	99.25 (96.05–100.85)	0.21 (-2.28–1.28) n.s.	99.08 (96.05–99.92)	0.25 (-1.87–2.27) n.s.
$D_{98\%, elective CTV}$	99.55 (97.82–103.33)	99.93 (98.52–101.16)	-0.37 (-1.66–2.44) p<.001	99.31 (98.51–101.94)	-0.09 (-0.90–1.38) n.s.	99.65 (98.24–100.93)	-0.02 (1.29–3.09) n.s
$D_{2\%,CTV, all}$	103.30 (101.51–105.35)	102.51 (101.51–104.25)	0.68 (-0.70–2.14) p<.001	103.32 (101.25–105.36)	-0.04 (-1.53–1.97) n.s.	102.43 (101.05–104.77)	0.64 (-1.08–2.79) p<.001

Table 4. Average DICE, mean and max DTA for deformed structures of 6 different virtual phantom.

ROI	DICE coefficient	Mean DTA [cm]	Max DTA [cm]
CTVT	0.97	0.04	0.35
CTVN	0.95	0.05	0.32
CTV elective	0.95	0.05	0.45
GTVT	0.89	0.10	0.49
GTVN	0.88	0.05	0.46
Larynx	0.94	0.07	0.41
Mandible	0.95	0.04	0.41
Oral Cavity	0.96	0.06	0.39
Parotid	0.94	0.06	0.42
Spinal cord	0.95	0.03	0.32
Esophagus	0.87	0.06	0.36

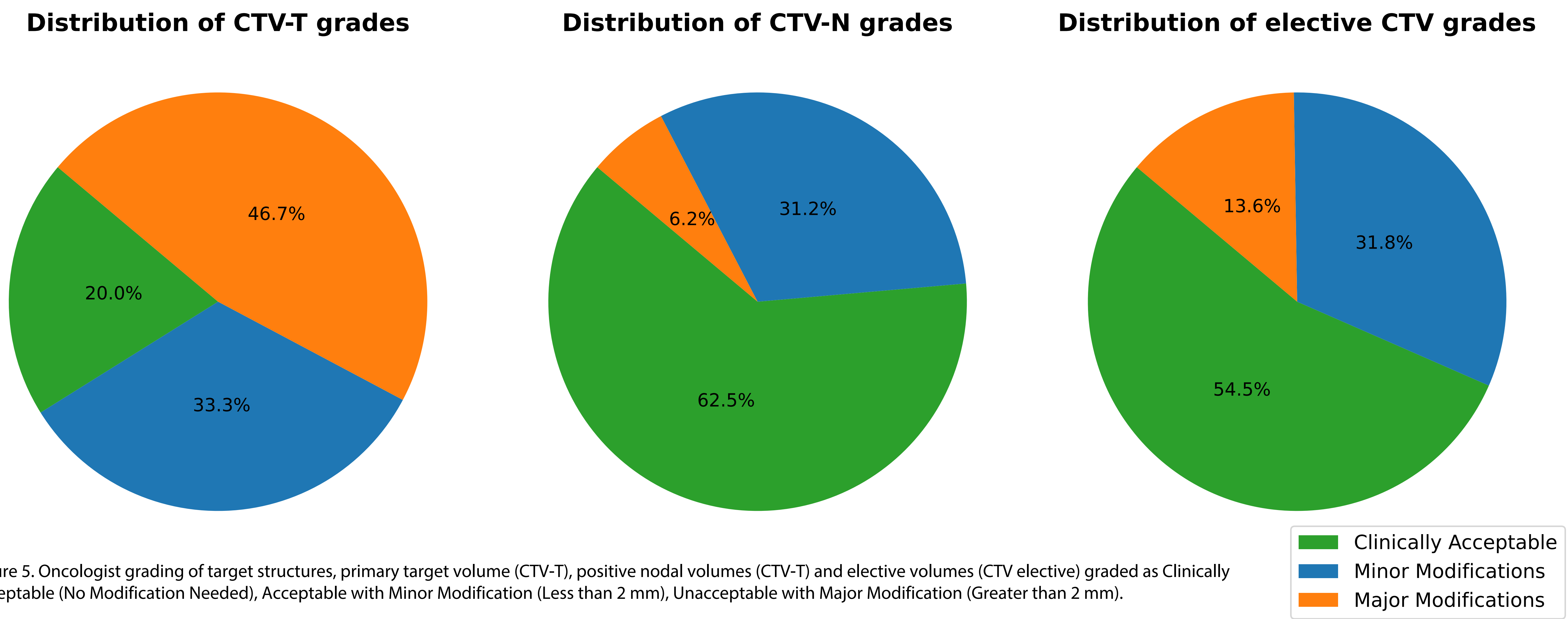

Figure 5. Oncologist grading of target structures, primary target volume (CTV-T), positive nodal volumes (CTV-T) and elective volumes (CTV elective) graded as Clinically Acceptable (No Modification Needed), Acceptable with Minor Modification (Less than 2 mm), Unacceptable with Major Modification (Greater than 2 mm).

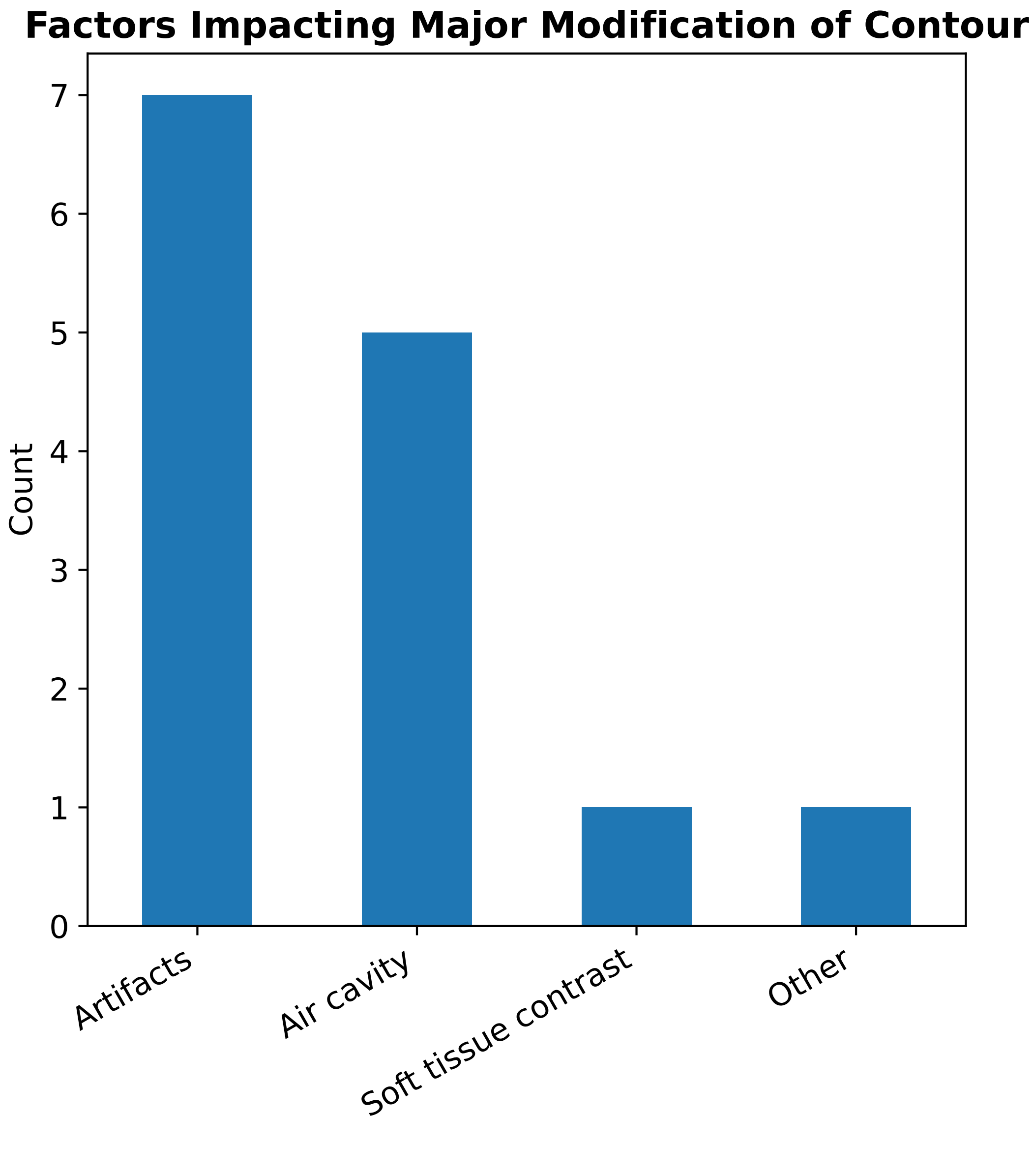

Figure 6. Factors impacting contours graded with Major Modifications. Oncologist have noted dental artifacts, changed volumes of air cavities and poor soft tissue contrast as factors impacting the integrity of contours.
